# Trends in medical students’ stress, physical, and emotional health throughout training

**DOI:** 10.1080/10872981.2019.1709278

**Published:** 2020-01-04

**Authors:** Isla McKerrow, Patricia A. Carney, Holly Caretta-Weyer, Megan Furnari, Amy Miller Juve

**Affiliations:** a Oregon Health & Science University, Portland, OR, USA; b Department of Family Medicine, Oregon Health & Science University, Portland, OR, USA; c Department of Emergency Medicine, Stanford University School of Medicine, Palo Alto, CA, USA; d Department of Pediatrics, Oregon Health & Science University, Portland, OR, USA; e Department of Anesthesiology & Perioperative Medicine, Oregon Health & Science University, Portland, OR, USA

**Keywords:** Basic science education, clinical science education, student wellness, curricular reform, curriculum evaluation, trainee health and wellness

## Abstract

**Background**: Medical student wellness, including physical health, emotional health, and levels of perceived stress, appears to decline during training, with students reporting high levels of depression, anxiety, and burnout as early as the first year of medical school. The impact of curricular changes on health and stress remains unclear, and a modified curriculum that compresses training of the foundational sciences and its effect on wellness has not been studied. Oregon Health & Science University School of Medicine has recently instituted a unique competency-based model, which provides an important opportunity to assess the effects of curricular change on student wellness.

**Objective:** Assess the effects of curricular change on student wellness.

**Design:** Medical students at a single institution were administered the SF-8, an 8-item health-related quality of life survey, as well as the Perceived Stress Scale, a 10-item scale that measures the degree to which life situations are appraised as stressful, at baseline (matriculation) and at the end of Year 1, 2 and 3. Individual variables were assessed over time, as well as a trend analysis of summary domain scores over the 4 time periods.

**Results**: Physical, emotional, and overall health were highest at baseline and lowest at the end of Year 1, after which they improved but never again reached baseline levels. Physical health declined less than emotional health. Perceived stress levels did not change over time but remained moderately high. There were no differences in health or perceived stress based on demographic variables.

**Conclusions**: In a competency-based curriculum, physical, emotional and overall health significantly worsened during Year 1 but improved thereafter, while perceived stress remained unchanged. Early in training, stress and poor overall health may be related to concerns about self-efficacy and workload. Although advanced students show improved wellness, concerns remained about emotional difficulties, such as anxiety and irritability, and feeling a lack of control.

## Introduction

As medical education strives to produce competent, well-rounded physicians, promoting medical student wellness has received increased attention [1–3]. ‘Wellness’, a broad term that encompasses physical and mental health, declines during training with worrisome implications for burnout as well as life satisfaction [4,5]. Prior studies suggest that mental health is especially affected, with higher levels of anxiety, depression, and perceived stress among medical students compared to age-matched non-medical student peers [6,7]. Boni et al. [8] found that over half of medical students experienced high cynicism and high emotional exhaustion in the early years of training. Matriculating medical students reported lower levels of burnout and depression symptoms compared to similarly aged college graduates [9]. However, within the first year of school, medical students reported higher-than-average levels of these same symptoms [9]. Although data on physical health outcomes are limited in the current literature, it too appears to worsen during training [10].

Although the literature suggests that mental or emotional and physical wellness are negatively affected by training, it has been more difficult to elucidate specific trends. School year appears to play a role, with the first and third years associated with higher levels of burnout and decrease in satisfaction, while fourth year is associated with greater resilience [8,11,12]. Effects of gender remain unclear. Some studies report higher stress levels in men [13], while others report disproportionate indicators of stress in women [14,15], and still others report no gender difference [7].

The impact of curricular changes on wellness remains unclear as well, and a modified curriculum that compresses training of the foundational sciences and its effect on wellness has not been studied. Slavin et al. [16] found that major curricular changes lead to ‘significantly lower levels of depressive symptoms, anxiety symptoms, and stress and significantly higher levels of community cohesion’, although these improvements were noted specifically in students who participated in a wellness program that preceded the curricular changes. The effects of curricular change on student wellness vary widely. Tucker et al. [11] found that the switch from discipline-based to systems-based courses lead to a worsening of quality of life, general stress, and poorer health. Contrarily, Kiessling et al. [17] examined effects of a ‘reformed’ curriculum that focused on small-group, problem-based learning and found that students in this reformed curriculum felt better supported ‘in terms of study conditions, social support at university, perceptions of their own attitudes and competencies, and living conditions’.

**Figure 1.**
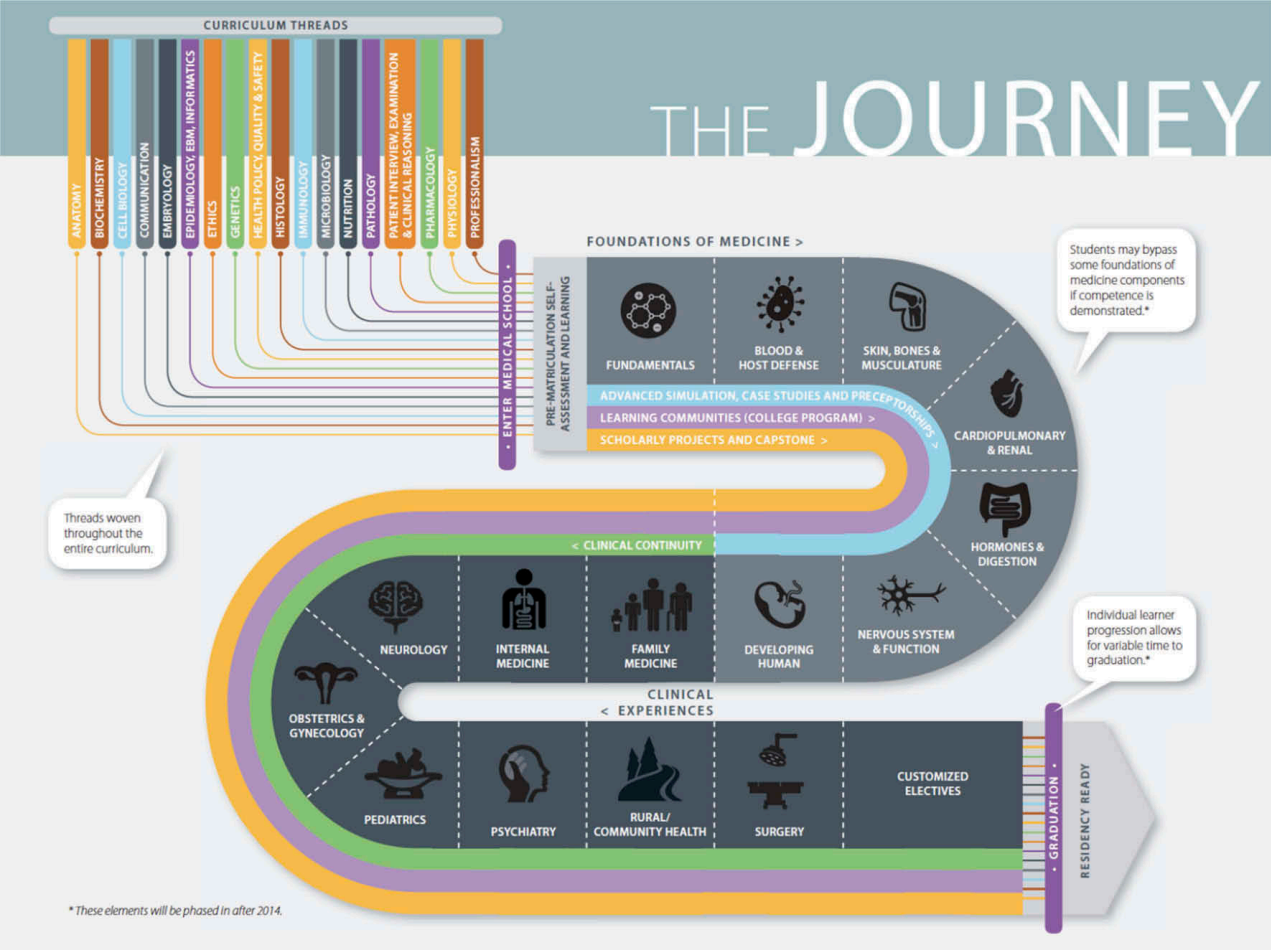
Depiction of OHSU YourMD curriculum including systems-based preclinical courses, seven core clinical experiences, customized clinical electives, and curricular threads woven throughout [33]

Recently, Oregon Health & Science University underwent a significant curriculum reform, with the resulting curriculum being a time-varying competency-based curriculum. In the new program, the preclinical years focus on an organ systems-based didactic approach with emphasis on small-group, problem-based learning through ‘Clinical Skills Labs’ as well as an early introduction to patient care through a preceptorship program. Woven throughout are curricular ‘threads’, which are content areas such as Anatomy and Ethics that are included longitudinally throughout the curriculum and are assessed within each system-based block (Figure 1). Assessments include frequent progress testing rather than traditional, higher-stakes exams. The preclinical foundational science phase of the curriculum is shortened to 18 months, with students completing didactics in December of the second year. Most students take Step 1 before February of the second year, and subsequently begin clinical rotations.

These curricular changes provide an important opportunity to assess medical student wellness in a unique competency-based curricular model which has not been reported in prior literature. In addition, the literature on physical health outcomes throughout training is currently sparse, and few other studies have assessed a student cohort over multiple time points. In this paper, we present the results of a longitudinal prospective cohort study tracking physical health, emotional health and perceived stress levels throughout the first 3 years of training in a recently revised, competency-based time-varying curriculum. By providing a set of sequential data points assessing overall health and stress levels, we aim to provide a robust picture of physical and emotional health trends throughout training in this new curricular style.

## Methods

### Participants

Medical students who matriculated to Oregon Health & Science University (OHSU) in August 2015 were included in this study. The students represented the second cohort to undertake the new time-variable, competency-based curriculum at OHSU [18,19] and were the first cohort from which we collected comprehensive data regarding both their demographic and wellness information. OHSU’s institutional review board approved all study activities (IRB #10873), which included notifying incoming participants that wellness surveys would be administered at designated intervals and that their participation, while encouraged, was voluntary. One hundred and forty-six medical students matriculated in August 2015, which provided the sample for this study.

### Instruments and data collection

Matriculating students received a survey in July 2015, prior to arriving at OHSU, that asked them their demographic information, including age, gender identity, race, ethnicity, marital status, parental status, state of residence, whether they were entering medical school immediately after completing undergraduate training, and which educational program they were enrolled in (MD only; MD/MPH; MD/PhD). Results from this survey are shared with the class during orientation so they have a sense of their cohort characteristics.

The wellness surveys we administered included the SF-8 and the Perceived Stress Scale [20,21]. The SF-8 is an 8-item health-related quality of life survey that assesses three health domains: overall health (assessed as a single-item), emotional health, and physical health. The Perceived Stress Scale (PSS) is a 10-item scale that measures the degree to which situations in one’s life are appraised as stressful, such as how unpredictable, uncontrollable, and overloaded respondents find their lives [21]. These surveys were administered at baseline, which occurred during orientation to OHSU’s medical school and before classes started, and then again at the end of Year 1 (May/June 2016), end of Year 2 (May/June 2017) and the end of Year 3 (May/June 2018). Response rates are typically so low after match day that the wellness survey was not administered a final time, given only 14 responses to a similar survey in the 4th year class that graduated in 2018 were received.

### Data analyses

The SF-8 uses five different response scales that range from 1 to 6 or 1 to 5 responses (e.g., 1 = Very poor; 2 = Poor; 3 = Fair; 4 = Good; 5 = Very good; 6 = Excellent; 1 = Not at all; 2 = Very little; 3 = Somewhat; 4 = Quite a lot; 5 = Could not do). We analyzed changes in individual variables across the four time points and then did the same with the domain scores (physical, emotional, and overall health). To calculate the physical and emotional health domain scores, we reverse scored all negative items toward a positive response and the scales were normalized to one of 1–100% to create equally weighted responses across the survey as described in prior scoring literature [22].

The PSS uses the same 5-point scale for all responses (1 = Never; 2 = Almost never; 3 = Sometimes; 4 = Fairly often; 5 = Very often). As with the SF-8, we analyzed individual variables of the PSS across the four time points. We reverse scored positively stated items, summed all items, and converted the total responses to a 1–100 scale. This allowed us to see how increases in perceived stress may be related to physical and emotional health over time.

Because our response rates decreased over time from 92.5% to 43.8%, we elected to conduct an unpaired analysis to optimize the statistical power we had at the four time points. One-way analysis of variance mixed model was used to assess individual variables over time. To account for multiple comparisons, we set the alpha level at p = 0.001 for statistical significance and all tests were two-sided. We conducted a trend analysis with summary domain scores over the four time periods. Statistical analyses were performed using SPSS Version 25.

## Results

Participants from the matriculating class of 2015 were predominantly female (54.2%), white (76.0%), not of Hispanic origin (95.9%), single (76.7%) and not parents (96.6%) (Table 1). Nearly 40% (39.5%) of participants were Oregon residents, the majority were in the MD only program (91.8%) and 10.3% were entering medical school directly from their undergraduate programs (Table 1).

**Table 1.** Characteristics of medical students at time of matriculation

	Med 2019 students
**Demographic characteristics**	n = 146
Mean Age in Years (SD*) Range	26.1 (3.5) 21–41
	n (%)
*Sex* Male Female	66 (45.2%) 79 (54.1%)
*Race* White Asian Native Hawaiian/Pacific Islander Black or African American American Indian/Alaska Native Other	111 (76.0%) 25 (17.1%) 0 3 (2.1%) 1 (0.7%) 6 (4.1%)
*Ethnicity* Non-Hispanic Hispanic or Latino	140 (95.9%) 6 (4.1%)
*Marital Status* Single (never married) Married/Partnered Separated Divorced Widowed	112 (76.7%) 29 (19.9%) 1 (0.7%) 4 (2.7%) 0
*Have Children* Yes No	5 (3.4%) 141 (96.6%)
*Oregon Resident* Yes No	57 (39.5%) 87 (60.4%)
**Educational characteristics**	n (%)
*Entered Medical School Right After Undergraduate Training Complete* Yes No	15 (10.3%) 131 (89.7%)
*Educational Program* MD Only MD/MPH MD/PhD	134 (91.8%) 7 (4.8%) 5 (3.4%)

†SD = Standard Deviation.

Individual variables reflecting overall health as well as physical and emotional health variables assessed across the four time points are presented in Table 2. We found no statistical differences over time according to demographic variables. Overall health was highest at baseline (mean = 4.71, standard deviation = 0.09), before entering the curriculum and was lowest at the end of Year 1 (mean = 4.07, standard deviation 1.1). Two physical health variables differed significantly across time periods (difficulty doing daily work, both at home and away from home because of physical health; and physical health or emotional problems that limited usual social activities with family or friends), with the highest physical health scores at baseline and the lowest scores at the end of Year 1 (Table 2). Two physical health variables did not change significantly. All emotional health variables were statistically different across the four time periods, with highest at baseline and lowest at the end of Year 1 (Table 2). Negatively scored PSS variables were lowest at baseline and highest at the end of Year 1 and positively scored variables reflected the inverse of this (Table 3). All but two of the PSS variables were statistically significant.

**Table 2.** SF-8 results: physical and emotional health scores according to the time point in the curriculum*

	2019 cohort				
	August 2015 (baseline) n = 132	May/June 2016 (end of year 1) n = 135	May/June 2017 (end of year 2) n = 115	May/June 2018 (end of year 3) n = 64	
Physical & emotional health variables	Mean (SD)	Mean (SD)	Mean (SD)	Mean (SD)	p value
**Overall health†**					
Overall, how would you rate your health during the past 4 weeks?	4.71 (0.9)	4.07 (1.1)	4.17 (1.2)	4.43 (1.5)	<0.001
**Physical health** **during the past 4 weeks …**					
How much did physical health problems limit your usual physical activities, such as walking or climbing stairs? ††	1.40 (0.7)	1.64 (0.9)	1.48 (0.7)	1.50 (0.7)	0.08
How much difficulty did you have doing your daily work, both at home and away from home, because of your physical health? ††	1.21 (0.5)	1.56 (0.8)	1.51 (0.8)	1.21 (0.6)	<0.001
How much did your physical health or emotional problems limit usual social activities with family or friends? ††	1.42 (0.7)	2.07 (1.1)	1.83 (1.0)	1.57 (0.9)	<0.001
How much bodily pain have you had?†††	1.86 (0.9)	2.07 (1.0)	2.24 (1.1)	2.07 (1.1)	0.29
**Emotional health** **during the past 4 weeks …**					
How much did personal or emotional problems keep you from doing your usual work, school or other daily activities?††	1.30 (0.5)	2.08 (1.0)	1.72 (1.0)	1.43 (0.9)	<0.001
How much energy did you have?††††	3.83 (0.7)	3.20 (0.9)	3.31 (0.9)	3.64 (0.8)	<0.001
How much have you been bothered by emotional problems, such as feeling anxious, depressed or irritable?†††††	2.02 (0.8)	2.70 (1.0)	2.43 (0.9)	2.89 (0.9)	<0.001

*Note scale changes.

†Scale: 1 = Very poor; 2 = Poor; 3 = Fair; 4 = Good; 5 = Very good; 6 = Excellent.

††Scale: 1 = Not at all; 2 = Very little; 3 = Somewhat; 4 = Quite a lot; 5 = Could not do.

†††Scale: 1 = None; 2 = Very mild; 3 = Mild; 4 = Moderate; 5 = Severe; 6 = Very severe

††††Scale: 1 = None; 2 = A little; 3 = Some; 4 = Quite a lot; 5 = Very much.

†††††Scale: 1 = Not at all; 2 = Slightly; 3 = Moderately; 4 = Quite a lot; 5 = Extremely.

**Table 3.** Perceived Stress Scale results: Perceived Stress Scores† according to time point in the curriculum

Perceived stress variables	August 2015 (baseline) n = 132	May/June 2016 (end of year 1) n = 135	May/June 2017 (end of year 2) n = 115	May/June 2018 (end of year 3) n = 64	
In the last month …	Mean (SD)	Mean (SD)	Mean (SD)	Mean (SD)	p value
How often have you been upset because of something that happened unexpectedly?	2.08 (0.7)	2.53 (0.9)	2.29 (0.9)	2.57 (1.1)	<0.001
How often have you felt that you were unable to control the important things in your life?	1.93 (0.8)	2.70 (1.1)	2.49 (1.0)	2.36 (0.8)	<0.001
How often have you felt nervous and ‘stressed’?	2.73 (0.9)	3.55 (1.0)	3.13 (0.9)	3.43 (1.2)	<0.001
How often have you felt confident about your ability to handle your personal problems?	4.27 (0.9)	3.87 (1.0)	3.89 (1.1)	4.29 (0.7)	0.002
How often have you felt that things were going your way?	4.14 (0.7)	3.57 (1.0)	3.56 (0.9)	4.21 (0.7)	<0.001
How often have you found that you could not cope with all the things that you had to do?	1.74 (0.7)	2.39 (1.2)	2.27 (1.1)	1.93 (0.9)	<0.001
How often have you been able to control irritations in your life?	4.10 (0.8)	3.45 (1.1)	3.7 (1.1)	4.00 (0.9)	<0.001
How often have you felt that you were on top of things?	4.08 (0.7)	3.16 (1.2)	3.44 (1.0)	3.64 (0.8)	<0.001
How often have you been angered because of things that were outside of your control?	2.08 (0.7)	2.55 (1.1)	2.29 (1.1)	2.43 (1.1)	0.001
How often have you felt difficulties were piling up so high that you could not overcome them?	1.64 (0.8)	2.41 (1.2)	2.01 (1.0)	1.79 (1.0)	<0.001

†Scale: 1 = Never; 2 = Almost never; 3 = Sometimes; 4 = Fairly often; 5 = Very often.

**Figure 2.**
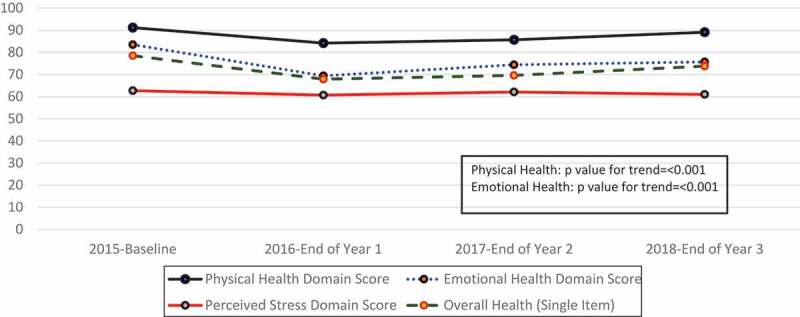
Normalized summary domain scores for physical health, emotional health, overall health, and perceived stress. The physical health domain score is a summary of items 2–5 of the SF-8 that specifically address physical health. The emotional health domain score is a summary of items 6–8 of the SF-8 that specifically address emotional health, while the overall health is a single item (item 10 from the SF-8)

The trend analysis of normalized domains in the SF-8 (physical, emotional and overall health) and the PSS (Figure 2) shows that health domain scores were highest at baseline and lowest at the end of Year 1. After this time, they gradually decreased or rose, respectively. By the end of Year 3, they were the closest to baseline that they would ever achieve, with the data we included in these analyses (Figure 2). The trend analysis revealed significant differences over time for emotional, physical health, and overall health but did not note changes in perceived stress.

## Discussion

The goal of this study was to assess medical student wellness, as measured by physical health, emotional health, and perceived stress, from baseline through the end of the third year of training in an innovative competency-based curriculum. After performing longitudinal analyses, we found that physical, emotional, and overall health are highest at baseline, and reach their lowest point at the end of Year 1. These values then gradually improve over the course of training, although they never again reach baseline levels. Conversely, perceived stress levels showed no change over time and were moderately high at about 60 on a scale of 0 to 100. As a domain, physical health followed the same trend as overall health and emotional health domains, but students start training with higher levels of physical health than they did emotional health, and despite the downward trend, physical health levels remain consistently higher than emotional or overall health levels.

Significant differences exist between the new curriculum at our institution and traditional medical school curricula, including an abbreviated preclinical phase, a time-varying competency-based framework, frequent progress testing, and academic coaching, all designed to help our students achieve their full potential. In terms of time variability, 61 students (42%) in this class completed their program one term early. Similar curricular changes have shown varied effects on wellness, with some studies showing an overall negative benefit [11], while others show improved sense of support and self-assessed competence [17], features we did not include in our assessment. Because we do not have data from students in our prior traditional curriculum it is not possible to attribute our findings to either the new curriculum as designed or a sole component of the OHSU curriculum, such as its organ systems-based approach with interwoven threads over time that are relevant for each organ system. Nevertheless, our data show several interesting trends, especially when examining individual time points.

A striking trend in our data is the decrease in all health domains from baseline to the end of Year 1. Similarly, other studies found difficulties in the first year of training [8]. As students are adjusting to a new program that is rapidly paced along with the potential loss of support systems, this downtrend is perhaps unsurprising. While the normalized overall perceived stress scores did not increase during Year 1 or decrease thereafter, several individual variables did show significant variability, reaching their peak during the end of Year 1. Additionally, overall scores did consistently hover at about 60 on a scale of 0–100, indicating perceived stress was moderately high and persisted over time. While the PSS is not validated as individual items, trends seen in these items may provide insight into the different stressors students experience. Several variables relate to workload in particular, including how often students felt they were in control of important things in their lives and how often they felt on top of things. This suggests that while perceived stress is not the sole contributor to students’ worsening wellbeing, concerns about the ability to handle responsibilities may worsen overall health. As students theoretically gain more efficacy throughout years of training, these specific perceived stress variables improve.

Prior studies suggest that wellness continues to decline in advanced years of schooling, specifically with worsening burnout, empathy and compassion fatigue [23–26]. Our data instead showed overall improvement in all three health domains. On the SF-8, the only variable that significantly worsened during the third year was the emotional health question ‘How much have you been bothered by emotional problems, such as feeling anxious, depressed or irritable?’ This is in line with studies demonstrating higher levels of emotional exhaustion, depersonalization, and disengagement in more senior students [24–26]. The PSS showed similar outcomes. Some negative variables, such as being upset at things out of their control, were at their worst in Year 3. Meanwhile, positive variables, such as students’ ability to handle personal problems or feeling that things were going their way, improved so much they actually exceeded baseline values. These data suggest that while third-year students experienced an improvement in overall wellbeing and may have felt optimistic regarding their own abilities, they also experience emotional distress influenced by a lack of control and negative personal experiences. The timing of survey delivery plays a role here; delivered at the end of the third year, the surveys captured students after they had successfully completed all core clerkships. The completion of this daunting phase, combined with the increase in schedule flexibility generally seen in Year 4, may improve overall student health. However, this timing also captured students as they begin residency applications in earnest, with the concomitant financial and interpersonal stress, which could contribute to the worsening of anxiety and feeling a lack of control.

Our data did not show any significant differences in health domains or perceived stress based on any demographic variable, including gender. The impact of gender on physician-trainee health, wellness, and burnout has been difficult to parse out given conflicting studies [7,13–15]. Interestingly, the data on gender differences become clearer as the level of training increases, with studies demonstrating an increase in burnout among female attending physicians [27–29]. If no gender difference is evident at the undergraduate medical level, it begs the question when in training or practice this disproportionate burnout takes effect. However, in this study, it may be that students were continuously stressed uniformly across their demographics because the curriculum was so novel.

In contrast to the health domain scores, stress levels did not vary significantly throughout training. We are not aware of other studies that normalized this data to make direct comparisons across perceived stress and emotional and physical health. However, in comparing PSS raw scores at our measured time points to other literature using the PSS to evaluate medical student stress, we find that PSS scores in our study are similar to those described elsewhere, if not slightly lower at baseline [30,31].

Normalized domain data shows improvement after the end of Year 1, but does not ever regain baseline levels, suggesting either that the decline in Year 1 is too substantial to recover from, that negative health effects persist and limit the extent that students can recover, or that programs do not offer enough targeted support during this year to help students deal with stress and undertake wellness activities. Given earlier data showing that students begin medical school with lower levels of burnout, and depressive symptoms than age-similar peers [9], this raises concern that training itself is detrimental to students’ health.

Strengths of this study include our comprehensive longitudinal within cohort design, which allowed us to focus on the effects of year of training, knowing that demographic variables did not vary. In addition, we used robust quality of life and stress measures which have validity evidence to support their use in similar settings and populations [21,22,32]. Limitations of this study include lower response rates over time (92.5% to 43.8% from baseline to end of Year 3) which prevented paired analysis. Additionally, surveys were completed only once yearly as opposed to multiple times throughout each year, which was not feasible due to time and resource limitations and survey fatigue among students. This study was conducted at a single institution and the cohort followed in this study was only the second cohort to matriculate into the new time-varying competency-based curriculum. Given the differences between the OHSU curriculum and traditional curriculum, especially the differences in the timing of key stressors (i.e., USMLE Step 1), our findings are not generalizable to traditional curriculum students at other institutions; however, they provide novel information needed for comparisons to future research.

As we begin to more fully understand what wellness and stress look like throughout medical school, future study directions include examining interventions, including curricular changes and focused wellness programming, that reduce stress and improve physical and emotional health. This involves understanding which resources students access, at what time in training, and with what frequency they do so. Another area of study would be to understand what social support students have, including whether they have a strong community while in school, and the impact this has on wellbeing and stress. Finally, the relative sparing of physical health compared to emotional health may warrant further investigation to determine if this is a regional-specific finding or if it is replicated in other programs.
